# Kinetics of Catalyst-Free and Position-Controlled Low-Pressure Chemical Vapor Deposition Growth of VO_2_ Nanowire Arrays on Nanoimprinted Si Substrates

**DOI:** 10.3390/ma15217863

**Published:** 2022-11-07

**Authors:** Sergey V. Mutilin, Lyubov V. Yakovkina, Vladimir A. Seleznev, Victor Ya. Prinz

**Affiliations:** 1 Rzhanov Institute of Semiconductor Physics SB RAS, 13 Lavrentiev Aven., 630090 Novosibirsk, Russia; 2 Nikolaev Institute of Inorganic Chemistry SB RAS, 3 Lavrentiev Aven., 630090 Novosibirsk, Russia

**Keywords:** nanowires, vanadium dioxide, selective area, CVD, VO_2_, phase transition, growth process

## Abstract

In the present article, the position-controlled and catalytic-free synthesis of vanadium dioxide (VO_2_) nanowires (NWs) grown by the chemical vapor deposition (CVD) on nanoimprinted silicon substrates in the form of nanopillar arrays was analyzed. The NW growth on silicon nanopillars with different cross-sectional areas was studied, and it has been shown that the NWs’ height decreases with an increase in their cross-sectional area. The X-ray diffraction technique, scanning electron microscopy, and X-ray photoelectron spectroscopy showed the high quality of the grown VO_2_ NWs. A qualitative description of the growth rate of vertical NWs based on the material balance equation is given. The dependence of the growth rate of vertical and horizontal NWs on the precursor concentration in the gas phase and on the growth time was investigated. It was found that the height of vertical VO_2_ NWs along the [100] direction exhibited a linear dependence on time and increased with an increase in the precursor concentration. For horizontal VO_2_ NWs, the height along the direction [011] varied little with the growth time and precursor concentration. These results suggest that the high-aspect ratio vertical VO_2_ NWs formed due to different growth modes of their crystal faces forming the top of the growing VO_2_ crystals and their lateral crystal faces related to the difference between the free energies of these crystal faces and implemented experimental conditions. The results obtained permit a better insight into the growth of high-aspect ratio VO_2_ NWs and into the formation of large VO_2_ NW arrays with a controlled composition and properties.

## 1. Introduction

Vanadium dioxide (VO_2_) is a highly correlated electronic material widely studied due to the reversible insulator-metal phase transition (IMT) occurring at ~68 °C [1,2,3,4]. During the phase transition, the VO_2_ crystal lattice changes from a low-temperature monoclinic insulating (I) phase to a high-temperature tetragonal metallic (M) phase. Simultaneously, there occurs a sharp change in optical reflectivity [5,6], a drop in resistivity amounting to several orders of magnitude [7], and a change in the lattice constant up to 1% [8]. Various devices such as optical switches [9], smart windows [10,11,12,13], Mott transistors [14,15], memristors [16,17,18], sensors [19,20], thermal actuators [21,22,23], etc. have been developed on the basis of the phase-transition phenomenon in VO_2_. However, the mechanical stresses arising at the grain boundaries and dislocations, together with stoichiometry fluctuations, lead to the extension of both the temperature range in which the phase transition in VO_2_ is observed, and the width of the transition hysteresis and they also considerably reduce the durability of the formed devices [24,25]. This circumstance seriously hampers the polycrystalline VO_2_ film integration into the industrial technology. Thin VO_2_ films can be replaced with single-crystal VO_2_ nanowires (NWs), which, as a rule, present high-quality, single-domain VO_2_ nanocrystals exhibiting a very sharp phase transition with a narrow temperature hysteresis not exceeding 1–2 °C [26]. Vertical VO_2_ NWs are stable, with respect to the crystal–substrate interface deformations that occur due to the phase transition. Successful applications of the single-domain phase transition in VO_2_ NWs have been demonstrated in many VO_2_-based devices such as actuators [23,27], gas sensors [28], power meters [29], strain gauges [30], etc. Such promising VO_2_ NW applications have attracted the considerable attention of researchers. However, for a wide practical application, a technology for the formation of ordered arrays of identical VO_2_ NWs, which would be well compatible with silicon technology, is needed. Indeed, to develop functional devices based on VO_2_ NWs, it is important for researchers to have means to control the position, size, orientation, and microstructure of grown VO_2_ NWs so that large uniform VO_2_ NW arrays with controlled characteristics can be formed. In recent years, there has been rapid progress in developing methods for the synthesis of single high-quality VO_2_ NWs and their arrays. However, such structures are still located on the substrates stochastically, and they have random sizes and random spatial orientations [12,31,32,33,34,35,36,37,38,39]. In our recent work, we have described the synthesis of strictly periodic free-standing vertical high-quality VO_2_ NWs using the selective area chemical vapor deposition (SA-CVD) process implemented on nanoimprinted Si substrates [40]. A non-catalytic synthesis of VO_2_ NWs occurs on top of the Si nanopillars, which act as crystal nucleation centers. Using this method, we can fabricate nanowires of a required shape at predetermined positions on the substrate without using catalysts, and achieve a fairly good control of the growth process. Despite the results obtained, the influence of the process conditions and the sizes of the nucleation centers on the kinetics of VO_2_ NW growth as well as the related size effects in VO_2_ NWs observed during the non-catalytic growth processes still remain disputable. The understanding of these important inter-relations should provide the required key information about the spatially selective and catalyst-free VO_2_ NW growth.

In the present work, we investigated the effect of various synthesis parameters on the non-catalytic SA-CVD growth of vertical and horizontal VO_2_ NW arrays on nanoimprinted silicon substrates in the form of the ordered arrays of nanopillars and nanostrips. It is shown that the height of vertically oriented VO_2_ NWs decreases with an increase in the area on which such NWs selectively grow. Based on the material balance equation, we provide a qualitative description to the process of vertical VO_2_ NW growth. According to the differences in the growth process of high-aspect ratio VO_2_ NWs oriented perpendicularly and in parallel to the substrate surface, we demonstrate the existence of two limiting growth regimes for VO_2_ crystal faces with a high surface energy determining the conditions for the NW-apex growth and for the {011} VO_2_ lateral crystal faces with a minimum surface energy. In the former case, the growth is controlled by diffusion, and in the latter case, by the chemical reaction rate. In our experiments, this led to the fact that, during the growth of vertical VO_2_ NWs, their height increased linearly with the synthesis time, whereas the lateral dimensions remained roughly unchanged. As a result, an increase in the aspect ratio of the formed NWs was observed. We believe that our new results will prove useful in paving the way toward the development of novel metal-insulator transition-based devices using ordered VO_2_ NW arrays such as tunable metamaterials, photonic crystals, neuromorphic systems, etc.

## 2. Experimental

The periodically arranged VO_2_ nanostructures on Si(001) substrates were formed using large-area double-stamp nanoimprint lithography (NIL) and the anisotropic reactive ion etching of Si substrates in CF_6_ plasma [41]. A detailed description of the formation process of such nanostructures is described in the Supplementary Materials, Section S1. As a result, two types of nanostructures were formed on the initially flat silicon substrate surface: a periodic array of 150 nm high and 80 nm wide nanostrips with a 180 nm pitch, and a periodic array of 150 nm high nanopillars with an 80 × 80 nm^2^ square cross section. Double nanoimprint lithography implemented on large surface areas (nanostructures covered an area of more than 10 cm^2^) is a rather complicated technological process inevitably giving rise to pitch size fluctuations in random places on the substrate. This leads to the formation of single silicon nanopillars with different lateral sizes, which differ from the expected sizes of elements in the initial stamp of the 80 × 80 nm^2^ area on one substrate. We took advantage of this feature of the double NIL to investigate the impact of the transverse dimensions of the nanopillars and nanostrips on the formation and growth process of VO_2_ NWs.

Ordered arrays of free-standing single-crystal VO_2_ NWs were grown on a nanoimprinted silicon substrate using a low-pressure chemical vapor deposition (LP CVD) process implemented in a two-zone horizontal reactor at the pressure of 2 Torr and the temperature of 450 °C [40]. The precursor was 98% vanadylacetylacetonate (Aldrich). The precursor vapor was introduced into the reactor together with an argon flow and the volumetric flow rate was 130 sccm. Oxygen was used as an oxidizing agent that was continuously supplied into the reactor at the flow rate of 60 sccm. The synthesis duration varied from 60 to 300 min.

The synthesized VO_2_ NW morphology was examined on JEOL-ISM-6700F and Hitachi SU8220 scanning electron microscopes (SEMs) at electron-beam energies ranging from 2 to 5 kV. The VO_2_ NW crystal structure was investigated by means of X-ray diffractometric measurements performed on a Shimadzu XRD-7000 diffractometer (CuKα radiation, Ni filter, 2θ = 5–60°, step 0.03°, data accumulation time 1 or 5 s) in the θ–2θ regime. X-ray photoelectron spectroscopy (XPS) measurements were made using a SPECS GmbH ProvenX-ARPES system equipped with an ASTRAIOS 190 electron energy analyzer and 2D-CMOS electron detector. The excitation source was focused monochromatic Al Kα radiation (hv = 1486.7 eV, 0.5 mm X-ray spot FWHM at 150 W anode power). XPS spectra were recorded at normal emission and constant pass energy of 30 eV with a total energy resolution of ≤0.6 eV. The spectrometer energy scale was calibrated by setting the measured Ag3d5/2 line binding energy of 368.22 ± 0.05 eV with respect to the Fermi energy EF.

## 3. Results and Discussion

**Figure 1:**
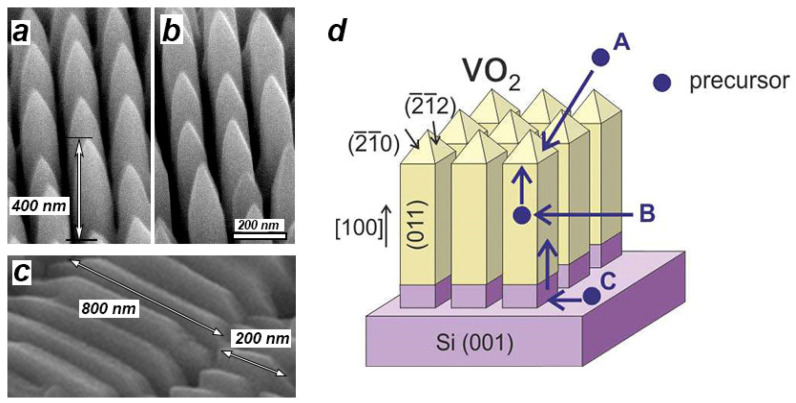
(**a**,**b**) Typical SEM images of the ordered array of free-standing vertical VO_2_ NWs grown on a Si substrate in the form of a square nanopillar array. The lateral dimensions of each NW were 80 × 80 nm^2^; they coincided with the lateral dimensions of the silicon nanopillars, their length being about 400 nm. (**c**) Typical SEM image of the ordered array of horizontal VO_2_ NWs grown on a Si substrate in the form of an array of 80 nm width nanostrips with a 180-nm pitch. The approximate lengths of horizontal NWs are indicated with arrows. The height of these NWs was almost independent of their contact area with the substrate. (**d**) Schematic representation of an array of vertical VO_2_ NWs. The purple arrows in (**d**) illustrate the mechanism of VO_2_ NW growth, which includes the direct precursor deposition onto the NW apex (A), diffusion of precursor species to the NW apex from the lateral crystal faces of VO_2_ NWs (B) and from the substrate surface through the lateral crystal faces (C).

Figure 1a–c shows typical SEM images of the ordered arrays of vertical and horizontal VO_2_ NWs formed under standard synthesis conditions during 120 min on the top of silicon nanopillars and nanostrips, respectively. Note that the lateral sizes of VO_2_ NWs were coincident with the lateral sizes of the flat apices of the silicon nanopillars and nanostrips. As we have shown earlier in [40], each element of the array presented a high-quality single-crystal VO_2_ nanocrystal of the monoclinic phase (space group P2_1_/c). High-aspect ratio VO_2_ NWs exhibited a preferred direction of their fastest growth corresponding to the [100] growth direction.

**Figure 2:**
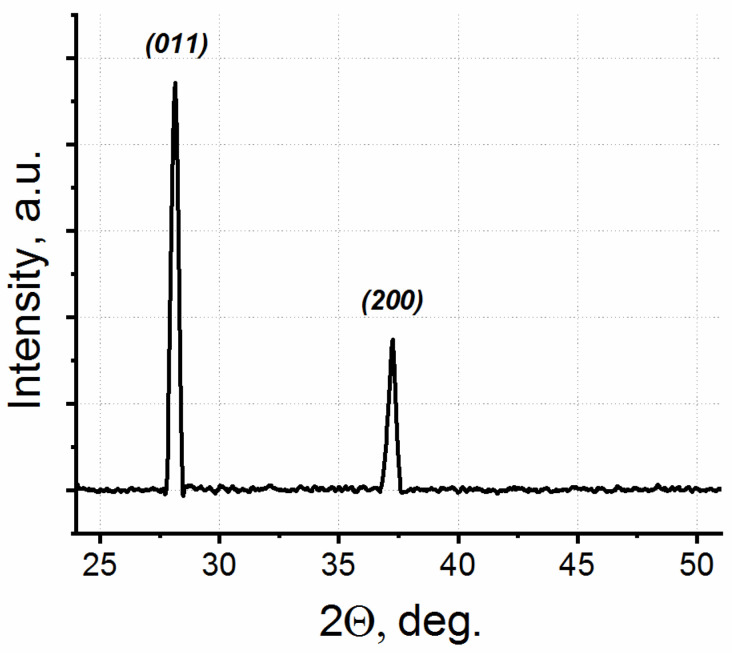
The X-ray diffraction spectrum of the horizontally and vertically oriented VO_2_ NWs synthesized on the nanoimprinted Si substrate.

Figure 2 shows a typical X-ray diffraction (XRD) spectrum with two pronounced peaks. These peaks correspond to (011) and (200) peaks in monoclinic VO_2_. No peaks due to other phases or impurities were detected in the spectrum, pointing to a high phase purity of our VO_2_ nanostructures. Note that the (011) peak corresponded to horizontally oriented VO_2_ NWs, while the (200) peak corresponded to vertically oriented NWs. Since the beam area was about 1 cm^2^, the obtained spectrum included information from almost the entire sample containing both horizontally and vertically oriented NWs with different cross-sectional areas. The fact that only two peaks were present in the XRD spectrum indicates that the NWs were strictly oriented on the substrate. The intensity of the peak (011) was higher than the intensity of the peak (200), because the relative content of horizontally oriented NWs on the substrate was several times higher than that of the vertically oriented NWs; this was due to the peculiarity of the double-stamp NIL and was discussed in detail in [40].

**Figure 3:**
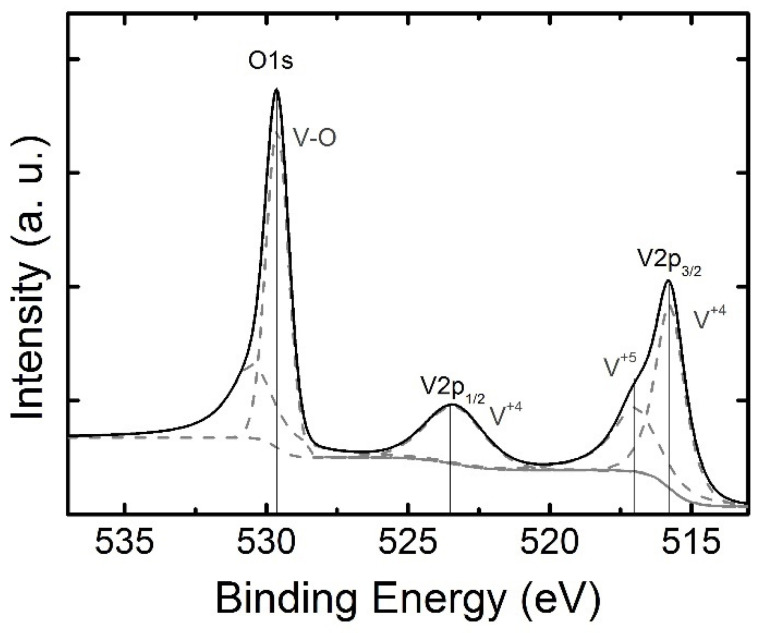
The XPS fine spectrum (O1s and V2p levels) of the VO_2_ NWs. Two peaks at 515.8 and 523.5 eV corresponded to V2p_3/2_ and V2p_1/2_ and showed the V^+4^ oxidation state. The presence of the V^+5^ component in the VO_2_ NWs denotes an over-oxidation, due to the existence of V_2_O_5_ at the surface of the VO_2_ NWs from air exposure.

In order to determine the elemental composition of the NWs and vanadium oxidation states, XPS spectra were obtained. The XPS survey spectrum of the VO_2_ NWs is given in Supplementary Material Figure S2. The XPS data were analyzed with the SpecsLab Prodigy software. Figure 3 shows the XPS fine spectrum (O1s and V2p levels) obtained from VO_2_ NWs. The energy peak at 529.6 eV relates to the binding energy of the O–V bonds. The V2p energy level splits into the V2p_1/2_ and V2p_3/2_ components due to orbital splitting. Two peaks at 515.8 and 523.5 eV correspond to V2p_3/2_ and V2p_1/2_ and show the V^+4^ oxidation state [42,43]. The deconvolution of the V2p_3/2_ peak exhibits two components corresponding to the V^+4^ and V^+5^ oxidation states. The presence of the V^+5^ component in the VO_2_ NWs denotes an over-oxidation due to the existence of V_2_O_5_ at the surface of the NWs from air exposure.

The synthesis of VO_2_ structures using CVD is a complex, multi-step and still insufficiently studied process [44]. The VO_2_ NW growth can either be due to the mechanism of diffusion growth or due to the direct chemical interaction of precursor species with the crystal surface. Since the preferred crystal growth direction was clearly traced in the experiments, the VO_2_ NW growth during CVD could involve the following processes (Figure 1d) [45]: (A) direct deposition of VO_2_ molecules from the precursor vapor, (B) diffusion of adparticles deposited onto the lateral surface of the VO_2_ NW to the top of the VO_2_ NW crystal, and (C) the diffusion of adparticles from the substrate surface to the top of the growing VO_2_ NW along the lateral walls.

**Figure 4:**
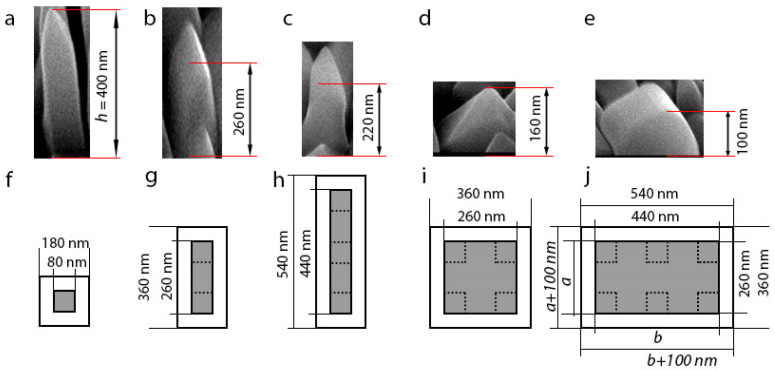
SEM images (side view) of the free-standing vertical VO_2_ NWs (upper part of the figure) grown on silicon nanopillars with different cross-sectional areas; the diagrams are shown in the lower part of the figure. SEM image and cross-sectional area of single Si nanopillar with: (**a**,**f**) square section of 80 × 80 nm^2^; (**b**,**g**) rectangular section of 80 × 180 nm^2^; (**c**,**h**) rectangular section of 80 × 440 nm^2^; (**d**,**i**) square section of 260 × 260 nm^2^, and (**e**,**j**) rectangular section of 260 × 440 nm^2^. In the schematic image, the gray area shows the surface of the silicon nanopillars on which the NWs grow; the light area around is the part of the substrate that was subject to etching during the formation of the periodic nanostructures and on which no crystal nucleation occurred.

Consider now the height of a vertical VO_2_ NW as a function of the cross-sectional area of the top of the silicon nanopillar on which the nucleation proceeds. As a result of NIL, each Si substrate contains several types of nanopillars differing in their transverse dimensions. Figure 4 shows the SEM images (side view) of individual vertical VO_2_ NWs with different lateral sizes. Shown below each SEM image is a schematic representation of the respective initial element on the nanostructured substrate (top view). The nanopillar region (cross-section of the formed VO_2_ NW) is highlighted in a gray color, and the outer light region is part of the etched silicon substrate on which no VO_2_ synthesis occurred. The regular single Si nanopillar had a square section of 80 × 80 nm^2^ (Figure 4a,f). Other Si nanopillars on the substrates had a rectangular section of 80 × 180 nm^2^; when two initial nanopillars merged together (Figure 4b,g), 80 × 440 nm^2^; formed by three initial nanopillars standing in a line (Figure 4c,h), 260 × 440 nm^2^; formed by six initial nanopillars arranged in an array of 2 × 3 pillars (Figure 4e,j) and a square section of 260 × 260 nm^2^; and formed by four original nanopillars arranged in a 2 × 2 pillar array (Figure 4d,i). The growth of single vertical single-crystal VO_2_ NWs was observed on such nanopillars. The grown vertical VO_2_ NWs had lateral dimensions coincident with the lateral dimensions of the silicon nanopillars. 

As is evident in Figure 4, the height of vertically growing VO_2_ NWs decreased when the cross-sectional area of such NWs increased in value. In other words, an inverse correlation was observed between the length of such VO_2_ NWs and their cross-sectional area. This finding points to the surface diffusion mechanism for the growth of such VO_2_ NWs, with VO_2_ adparticles being collected on the apex of NWs as they diffuse from the substrate and from the lateral crystal faces of NWs. The source of attraction for VO_2_ adparticles is, apparently, the top/upper crystal faces of VO_2_ NWs. A similar phenomenon was also observed during the selective synthesis of A_3_B_5_ NWs [46]. In the case in which the interaction between the precursor species and the VO_2_ crystal during the synthesis would occur exclusively on the crystal surfaces, vertical VO_2_ NWs with different transverse dimensions would then have identical heights.

In order to gain a better insight into the details of the VO_2_ NW growth process, we used an approximation to estimate the growth rate of vertical VO_2_ NWs based on the material balance equation and similar to that usually used to analyze the A_3_B_5_ NW growth (see, e.g., [47,48]). According to the material balance equation, the increase in the VO_2_ NW volume in the case of interest can be expressed as follows:

(1) $$a\cdot b\cdot \Delta h=\{C_{1}\cdot a\cdot b\cdot I_{VO2}\cdot \chi +C_{2}\cdot S_{LAT}\cdot I_{VO2}\cdot \chi^{\prime }\cdot (1-\varepsilon )+C_{3}\cdot S_{SUB}\cdot I_{VO2}\cdot \chi^{\prime \prime }\cdot (1-\varepsilon^{\prime })\}\cdot \Delta t$$

where *a* and *b* are the lengths of the VO_2_ NW lateral sides; Δ*h* is the increment of height *h* of the VO_2_ NW having grown during the synthesis time Δ*t*; *S_LAT_* is the lateral NW surface area (*S_LAT_* = 2·*h*_0_·(*a* + *b*)); *S_SUB_* is the area of the free region on the substrate where no crystal synthesis occurs (*S_SUB_* = 400·(*a* + *b* + 25)); *I_VO_*_2_ is the particle flux density (in nm^−2^·s^−1^); *χ*, *χ*′, and *χ*″ are the adsorption probabilities of VO_2_ molecules on top of the VO_2_ NW, on the lateral NW walls, and on the substrate, respectively; *ε* and *ε*′ are the parameters determining the reverse VO_2_ molecules flow from the top of the VO_2_ NW to its lateral walls and to the substrate, respectively; *C*_1_, *C*_2_, and *C*_3_ are time-independent constants characterizing the growth process and its kinetics. For simplicity, we assumed that each NW had the shape of a rectangular parallelepiped with a flat apex. The first, second, and third terms in the curly brackets of Equation (1) corresponded respectively to: (a) the direct deposition of VO_2_ species from the gas phase, (b) the VO_2_ species diffusion from the lateral VO_2_ NW surface to its top, and (c) the VO_2_ species diffusion from the substrate region around the VO_2_ NW, where no crystal growth occurs, to the top. The expression *S_SUB_* = 400·(*a* + *b* + 25) is nothing but the area of the light region in Figure 4 plus the lateral surface area of the 150 nm high silicon nanopillar. The VO_2_ NW height *h* as a function of time *t* can be derived by solving Equation (1); it contains the time-dependent factor *exp* {2·(*a* + *b*)·*t*/*a·b*}. As shown below, the vertical VO_2_ NW height dependence on the synthesis time can be approximated with linear dependence. On the assumption that Δ*h* << *h*, we can replace the height *h* in the second term in the curly brackets in Equation (1) with its constant value *h*_0_ and rewrite Equation (1) as

(2) $$\frac{\Delta h}{\Delta t}∼C_{1}\cdot I_{VO2}\cdot \chi +C_{2}\cdot \frac{2\cdot (a+b)}{a\cdot b}\cdot h_{0}\cdot I_{VO2}\cdot \chi^{\prime }\cdot (1-\varepsilon )+C_{3}\cdot \frac{400\cdot (a+b+25)}{a\cdot b}\cdot I_{VO2}\cdot \chi^{\prime \prime }\cdot (1-\varepsilon^{\prime })$$

This is a simplified expression for the vertical VO_2_ NW growth rate, which determines the NW height as a linear function of time.

**Figure 5:**
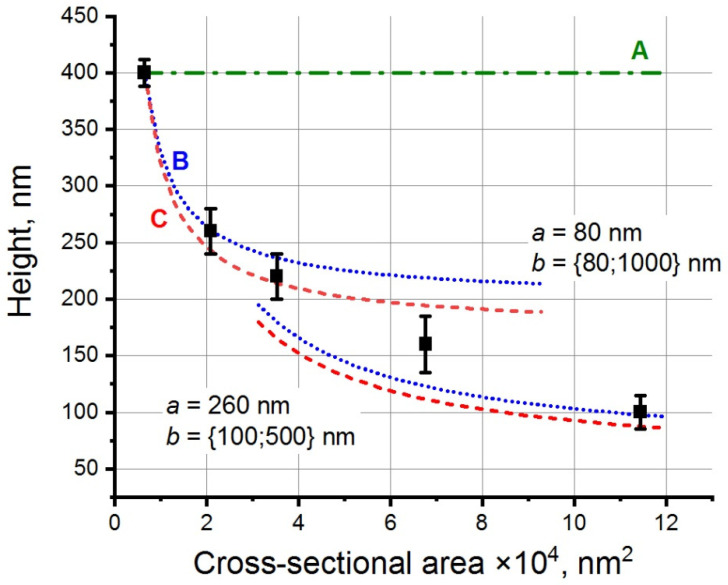
The height of vertical VO_2_ NWs as a function of their cross-sectional area. Black squares are the experimental points, straight line **A** (green dash dot) is the NW height dependence on the NW base area calculated on the assumption of the direct deposition of VO_2_ species from the gas phase, and curves **B** (blue short dot) and **C** (red short dash) are the dependences calculated by taking into account the diffusion of VO_2_ particles to the NW apices from the lateral surface of NWs and from the region of the substrate around the NWs, where there is no VO_2_ crystal growth.

Figure 5 shows the height dependence of vertical VO_2_ NWs on their cross-sectional area (black squares with error bars indicating the accuracy of determining the NW height). The calculated curves A, B, and C in Figure 5 were plotted under the assumption that the NW height is determined only by the contributions due to the first, second, and third term, respectively, in Equation (2).

Two types of curves B and C, which are plotted according to Equation (2) and differ in the NW lateral length *a* are shown in Figure 5. Indeed, the experimental points can be divided into two groups. The first group consists of three types of NWs, in which lateral length *a* is 80 nm, and lateral length *b* varies from 80 to 440 nm (see Figure 4f–h). The second group consists of NWs, in which the lateral length *a* is 260 nm, while lateral length *b* varies from 260 to 440 nm (see Figure 4i,j). Each group of experimental points is described by separate curves plotted according to Equation (2), in which the experimental values of the NW lateral length *a* equal to 80 or 260 nm, were substituted. The NW height dependences on the NW cross-sectional area shown with curves B and C were very similar to each other, in contrast to similar NW calculations for A_3_B_5_ materials (see, e.g., [46,47,48]). In the above articles, the NW height h as a function of the NW-base radius r shown with curve B varies as *1*/*r*, and with curve C—as *1*/*r*^2^, since the substrate surface area free of the crystal growth remains fixed, and only the NW-base radius is changed in its value. In our case, this area changed along with the change in the NW-base area (see Figure 4). The discontinuity in curves B and C shown in Figure 5 is related to the change in the VO_2_ NW side length *a* (whose value in the calculations was assumed constant) from 80 nm to 260 nm, while size *b* changed continuously from 80 nm to 500 nm. It is seen that the experimental points fell within the range between the calculated *h* values in curves B and C. The only exception was the point for VO_2_ NWs with the NW square section equal to 260 × 260 nm^2^. As seen in the figure, the shape of this crystal was more like a pyramid than a NW. A substantial contribution due to the pyramidal apex to the NW volume for such growth times leads to errors in the determination of this NW height, which was calculated as described above for the shape of a rectangular parallelepiped.

The experimental points in Figure 5 show that, to a large extent, the source of material for the vertical VO_2_ NW growth was the VO_2_ particle diffusion to the top of the NWs from their lateral surface and/or from the free substrate areas where no crystal synthesis occurred. It is not possible to separate out these two particle sources in experiments. However, none of the experimental data corresponded to curve A. This means that the direct deposition of VO_2_ particles from the gas phase is not the main factor for the vertical VO_2_ NW growth.

Consider now the growth process of horizontal VO_2_ NWs. These NWs are oriented along the direction [100] in the substrate plane, with the crystal face of such NWs directed along the normal to the substrate having the direction [011]. Similarly, in the case of vertical NWs, let us analyze Expression (2) for the vertical growth rate of such nanostructures.

As is evident in the SEM image in Figure 1c, in contrast to the vertically oriented VO_2_ NWs, the height of horizontal VO_2_ NWs is practically independent of the area of their contact with the substrate. That is, the experimental results show that, according to Expression (2), the determining factor in the crystal growth rate here is the first term (A), which in this case corresponded to the direct VO_2_ particle deposition from the gas phase. This suggests that different growth mechanisms operate for different VO_2_ NW faces. For vertical NWs, this is the diffusion-controlled growth, and for horizontal ones, the direct particle deposition from the gas phase. To gain a better insight into the reasons for the different growth behavior of VO_2_ NWs on the different crystal faces of VO_2_ NWs, we considered the growth rate dependence of the vertical and horizontal VO_2_ NWs on the precursor concentration in the gas phase and on the growth time.

First, we considered the effect of the precursor concentration near the substrate surface on the VO_2_ NW growth rate. This dependence can be established by varying the partial pressure of precursor vapor in the gas phase via changing the heating temperature of the precursor source. However, large changes in the partial precursor pressure can lead to a change in its ratio in the mixture with oxygen. According to the phase diagram [49,50,51], this, in turn, may affect the phase composition of the synthesized VO_2_ NWs. The variation in the partial precursor pressure in a narrow range of values is difficult to control using a small change in the precursor evaporation temperature. This is why, to study the effect, due to small changes in the precursor concentration, on the VO_2_ NW growth rate along the normal to the substrate surface, we analyzed the substrate regions located at different distances from the edge of the substrate holder. It is known that, in CVD processes held in horizontal reactors, a gas-phase boundary layer forms on the substrate surface; in this layer, the flow velocity is close to zero (see Supplementary Materials, Figure S3). The thickness δ of the boundary layer depends on the coordinate; as a rule, it increases when the gases move along the substrate holder [52]:

(3) $$\delta (x)={\left(\frac{\mu \cdot x}{\rho \cdot U}\right)}^{1/2}$$

In Formula (3), *µ* is the gas viscosity, *ρ* is the gas mixture density, and *U* is the gas flow velocity before the impact onto the substrate holder. The *x*-axis is directed along the gas flow, its coordinate being counted from the front edge of the substrate holder. Since the boundary layer thickness *δ*(*x*) increases along the length of the sample, the effective mass-transfer coefficient *h_G_* decreases in value:

(4) $$h_{G}=\frac{D_{G}}{\delta (x)}$$

In Formula (4), *D_G_* is the coefficient of precursor species diffusion in the gas phase across the thickness layer *δ*(*x*). The gas phase diffusion coefficient is proportional to the gas viscosity *µ*, to gas density *ρ*, *µ*/*ρ*, and it only moderately varies with temperature, typically as *µ*/*ρ*~*T^ξ^*, where 0.5 < *ξ* < 1.75.

A decrease in the effective mass-transfer coefficient leads to a reduced surface concentration in the precursor species:

(5) $$C_{S}=C_{G}\cdot {\left(1+\frac{k_{S}}{h_{G}}\right)}^{-1}$$

In Formula (5), *C_S_* is the near-surface concentration of precursor molecules in the gas phase, *C_G_* is the volumetric concentration of precursor species in the gas phase far from the sample, and *k_s_* is the rate of the chemical reaction on the surface.

**Figure 6:**
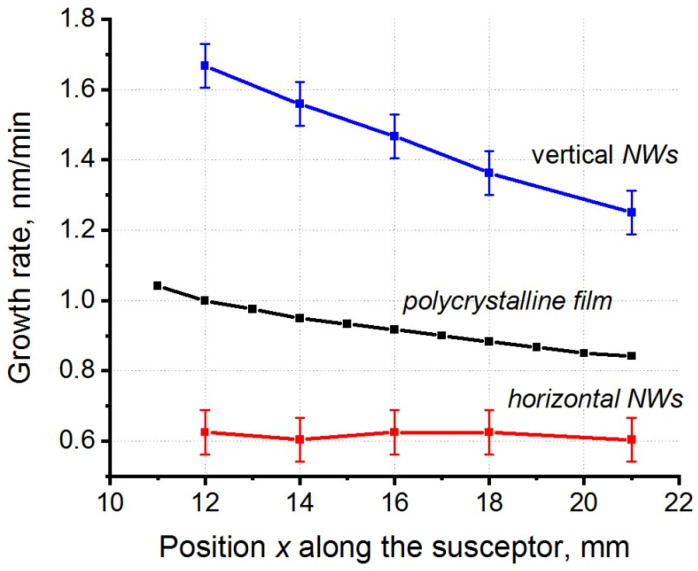
The growth rate of the VO_2_ NWs and a polycrystalline VO_2_ film as obtained by dividing the NW height by the synthesis time versus the location of NWs relative to the substrate holder edge. The gas flow is directed from left to right.

In addition, the gas phase undergoes a depletion with the precursor as the precursor propagates in the direction along the sample. Some of the precursor molecules are captured by the substrate, and their concentration in the gas phase moving along the substrate decreases in value. As a result, it can be observed in experiments that the growth rate of a deposited VO_2_ film decreases as the gas flow moves along the substrate holder. The profile of the growth rate along the substrate for CVD reactors with horizontal and inclined substrate holders is determined by the reactor geometry and by the synthesis conditions, and it can appear nonmonotonic [53]. In our experiments, the coordinate-nonuniform growth rate profile was determined from cross-section SEM images by analyzing the variation in the deposited film thickness of polycrystalline VO_2_ across the cleavage place, and this profile was shown to exhibit a monotonically decreasing behavior. The difference between the VO_2_ film growth rates at different points on the substrate was due to the variation in the near-surface precursor concentration along the substrate; under the adopted synthesis conditions, this difference was found to be about 20% over the length of 1 cm. Since, as shown above, the vertical NW growth rate depends on their cross-sectional area, in all further experiments, we considered only vertical NWs with a square cross section of 80 × 80 nm^2^. Figure 6 shows the growth rates of VO_2_ as dependent on the position on the substrate holder for polycrystalline VO_2_ films and for vertical and horizontal VO_2_ NWs. It can be seen from the graph that the vertical VO_2_ NW growth rate was large, and it decreased somewhat faster along the sample (33% over a length of 1 cm) than for a polycrystalline VO_2_ film. The vertical VO_2_ NW height was approximately twice the effective thickness of the deposited polycrystalline VO_2_ film. For horizontal NWs, the growth rate turned out to be almost independent of the position of the observation point on the substrate holder (or on the precursor concentration).

**Figure 7:**
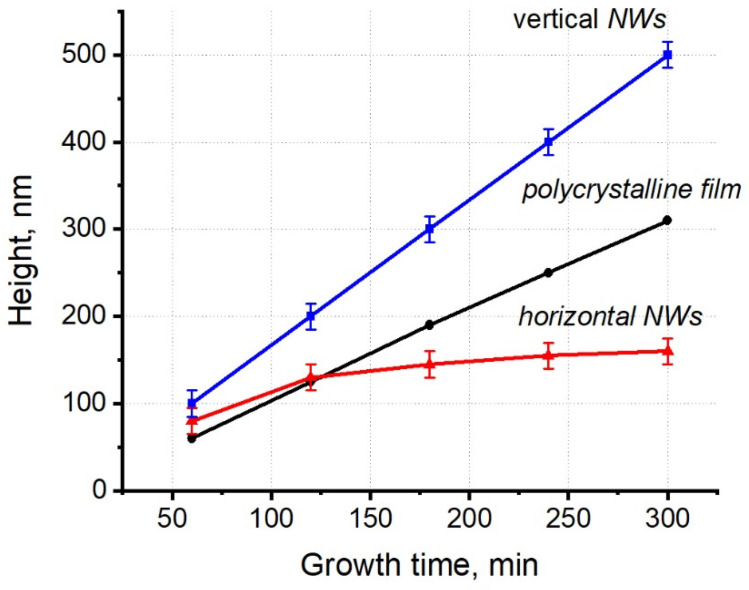
Dependence of the height of the VO_2_ NWs and a polycrystalline VO_2_ film on the synthesis time.

Consider now the total height of VO_2_ NWs as a function of the synthesis time. For comparison, Figure 7 shows the data for the vertical and horizontal NWs, and for a polycrystalline VO_2_ film. Evidently, both the height of vertical NWs and the film thickness can be approximated with a linear function of time. For horizontal VO_2_ NWs, the height increment measured along the normal to the substrate surface decays with time.

From Figure 6 and Figure 7, it follows that the VO_2_ NW growth in the [100] direction proceeds most rapidly, whereas the growth in the [011] direction demonstrates the lowest growth rate (both rates are determined by the synthesis conditions). From the dependence of the growth rate of horizontal NWs on the amount of consumed precursor material, it follows that the growth rate of {011} VO_2_(M) planes at a given synthesis temperature is limited by the rate of the chemical reaction (or, more specifically, by the rate of VO_2_ adparticle incorporation into the growing crystal). The excessive adparticle flux cannot be embedded into these surfaces and, as this flux increases, the amount of embedded VO_2_ particles per unit time remains roughly unchanged. In contrast, as seen in the experimental data, the growth rate along the [100] direction varied proportionally to the VO_2_ adparticle concentration.

For the polycrystalline VO_2_ films, due to the random orientation of its constituent NW crystals, we have an intermediate situation, with the growth rate of the VO_2_ film being dependent on the precursor concentration yet not as strongly as that of the similar dependence for the growth in the [100] direction. Such a profound difference in the growth of different crystal faces can be explained by the different values of their free surface energies. Indeed, the {011} crystal planes are the planes with the lowest surface energy and with the smallest number of free bonds on the surface. On the other hand, the {−2–10} planes, which form the NW apex of VO_2_ NWs growing in the [100] direction, have the highest surface energy (number of free bonds on these crystal faces). In Ref. [54], the surface energies were calculated for stable planes of equilibrium crystal morphology determined from the Wulff construction for the VO_2_ rutile phase. For the monoclinic phase, the planes with the minimum surface energy of 0.35 J/m^2^ turned out to be {011} planes, while those with the maximum energy of 1.6 J/m^2^—{−2–10} planes formed the top of VO_2_ NWs growing in the [100] direction. Thus, the VO_2_ NW growth patterns described above are characterized by the large difference between the surface energies of different VO_2_ (M) crystal faces. Indeed, the free surfaces of vertically oriented NWs are the surfaces of the fastest VO_2_ growth, and such NWs therefore exhibit an unlimited growth. The horizontally oriented NWs are limited by the lithographic pattern. Such NWs grow free along the substrate unless they meet a neighboring NW. In this case, the further NW growth in the [100] direction turns out to be limited and becomes retarded. Here, the VO_2_ nanowires can only grow in the directions normal to the {011} planes with a low growth rate.

In addition to the large difference in the surface energies on different crystal faces of VO_2_ NWs, other conditions are obviously important, leading to the growth of VO_2_ NWs with a large aspect ratio. One of the important parameters leading to the high-aspect structure growth is the diffusion length of VO_2_ adparticles on the surface of the VO_2_ crystal faces bounded by {011}-type planes. A large diffusion length of VO_2_ adparticles (greater than the NW length) leads to the migration of adparticles through the lateral surface of the VO_2_ NWs to their top, thus making the aspect ratio of the formed NW increase. A short diffusion length of VO_2_ adparticles led to slow VO_2_ NW growth in the lateral direction. As is known, one of the most important parameters determining the particle diffusion length is temperature. However, our experiments, in which the synthesis temperature was varied in a range from 400 to 500 °C, revealed no relation between the synthesis temperature and the aspect ratio of the grown VO_2_ NWs. However, the influence, due to the local temperature, on the lateral VO_2_ NW surface cannot be ruled out here. In the experiments, array elements (i.e., NWs) were spaced from one another by a distance of the order of their lateral sizes. Such an arrangement of NWs can lead to higher local temperatures on the lateral surfaces of the array NWs compared to single VO_2_ NWs, due to thermal coupling [55]. This small increase in temperature can slightly increase the VO_2_ adparticle diffusion length on the lateral VO_2_ NW surfaces, which in turn can result in the synthesis of high-aspect vertical NWs arranged in a dense ordered array.

## 4. Conclusions

In this work, we investigated the SA-CVD synthesis of VO_2_ nanowires, which is widely recognized as a promising method for the scaled-up formation of nanostructures and nanodevices. The results obtained provide insights into the processes of position-controlled, non-catalytic CVD growth of high-aspect VO_2_ NWs on nanoimprinted silicon substrates under controlled synthesis conditions. It was shown that the VO_2_ NW growth rate in the [100] direction decreased with an increase in the cross-sectional area of the VO_2_ NWs and increased with an increase in the precursor concentration. In this case, the NW height increased linearly with the synthesis duration, leading to an increased length-to-diameter aspect ratio. The NW length was about two times greater than the effective thickness of the deposited polycrystalline VO_2_ film, this being indicative of the diffusion growth nature. In contrast, the height of the horizontally growing VO_2_ NWs measured along the [011] direction does not depend on their cross-sectional area, nor does it depend on the precursor concentration. The increase in height slows down with an increase in the synthesis time, whose regularity is typical of reaction-rate-limited growth processes. Due to the different surface energies of the crystal faces that form the top of VO_2_ NWs and their lateral faces, and due to the large adparticle diffusion length over the substrate surface and over the lateral faces of the NWs, the SA-CVD synthesis yields high-aspect crystals, and this circumstance can be used to form vertical VO_2_-based heterostructures. Our results provide an important step toward the controlled growth of VO_2_ nanowire arrays required for the development of high-performance nanophotonic and nanoelectronic devices based on the vertical VO_2_ nanowires grown on the Si platform.

## Supplementary Materials

The following supporting information can be downloaded at: www.mdpi.com/article/10.3390/ma15217863/s1, Figure S1. The process of forming the nanostructured surface of a Si substrate using double-stamp nanoimprint lithography and reactive ion etching. After the first lithography and subsequent etching, arrays of periodic strips with a width of 80 nm, a pitch of 180 nm, and a height of 150 nm were formed on the substrate surface. After the second lithography and etching, arrays of square nanopillars with a width of 80 nm, a pitch of 180 nm, and a height of 150 nm were formed on the surface of the Si substrate. Figure S2. XPS survey spectrum of the VO_2_ NWs grown on a nanoimprinted Si substrate. Figure S3. Schematic representation of the diffusion process of precursor particles through the boundary layer in the gas flow. The boundary layer is shown with the dashed blue curve. The dependence δ(x) is the thickness of the boundary layer versus the distance from the edge of the substrate holder (sample).
