# Supplementary material for: Kinetics of Catalyst-Free and Position-Controlled Low-Pressure Chemical Vapor Deposition Growth of VO2 Nanowire Arrays on Nanoimprinted Si Substrates
Source: Materials (Basel). 2022 Nov 7;15(21):7863. doi: 10.3390/ma15217863 (PMC9656171; doi:10.3390/ma15217863)
Supplement: Supplementary file 1 [file materials-15-07863-s001.zip › materials-1985872-supplementary.pdf]

Supplementary Materials for

Kinetics of catalyst-free and position-controlled low-pressure chemical vapor deposition growth of VO_2 nanowire arrays on nanoimprinted Si substrates

S.V. Mutilin[1,*], L.V. Yakovkina[2], V.A. Seleznev[1], V.Ya. Prinz[1]

1 Rzhanov Institute of Semiconductor Physics SB RAS, 13 Lavrentiev aven., Novosibirsk, 630090, Russia
2 Nikolaev Institute of Inorganic Chemistry SB RAS, 3 Lavrentiev aven., Novosibirsk, 630090, Russia
* mutilin@isp.nsc.ru

Section S1. Nanostructured silicon substrates

For forming the arrays of Si nanopillars with a square cross-section of 80x80 nm^2 on the surface of a (001) Si substrate, double nanoimprint lithography was used. For this, a master stamp, which was a nanostructured Si substrate with a diameter of 150 mm, was used. The surface of the master stamp consisted of periodically extended nanogrooves 155 ± 5 nm deep and 100 nm wide with a pitch of 180 ± 5 nm. With its help, an intermediate polymer stamp was prepared, which then was used for combined UV treatment and thermal stamping of the 80-nm thick TU-2-90 resist on a flat silicon substrate. Schematically, the process of forming a nanostructured Si surface is shown in Figure S1. The resist stamping was carried out at a temperature of 80 ºC and a pressure of 50 bar for 5 minutes with its photopolymerization with ultraviolet light for 1 minute (Figure S1a). As a result of the first lithography, a periodic resist lattice with a strip width of 80 nm, a strip height of 150 nm, and a pitch of 180 nm was formed on the silicon surface (Figure S1b). Anisotropic reactive ion etching of the Si substrate in SF_6 plasma to a depth of approximately 150 nm was performed through the mask thus formed (Fig. S1c). Then, the resist residues were removed for 20 minutes in a solution consisting of 3 parts of H_2SO_4 per 1 part of H_2O_2 (Figure S1d).

As a result, a periodic array of nanogrooves with a depth of 150 nm and a width of 100 nm, with a pitch of 180 nm, was formed on the initially flat surface of the silicon substrate. Next, an 80-nm thick layer of TU-2-90 resist was deposited onto this substrate with nanogrooves, and the second nanoimprint lithography was performed. In this case, the nanogrooves on the polymer stamp were oriented at an angle of 90° with respect to the silicon nanogrooves on the substrate (Fig. S1e). This led to the formation of an array of square nanopads of the resist located on the top of silicon nanostrips (Figure S1e). During the subsequent reactive ion etching of the Si substrate, Si nanopillars with a 80x80-nm^2 square cross-section were formed (Fig. S1g). The resist was removed from the Si substrate in the same manner as it was made after the first lithography (Figure S1h).

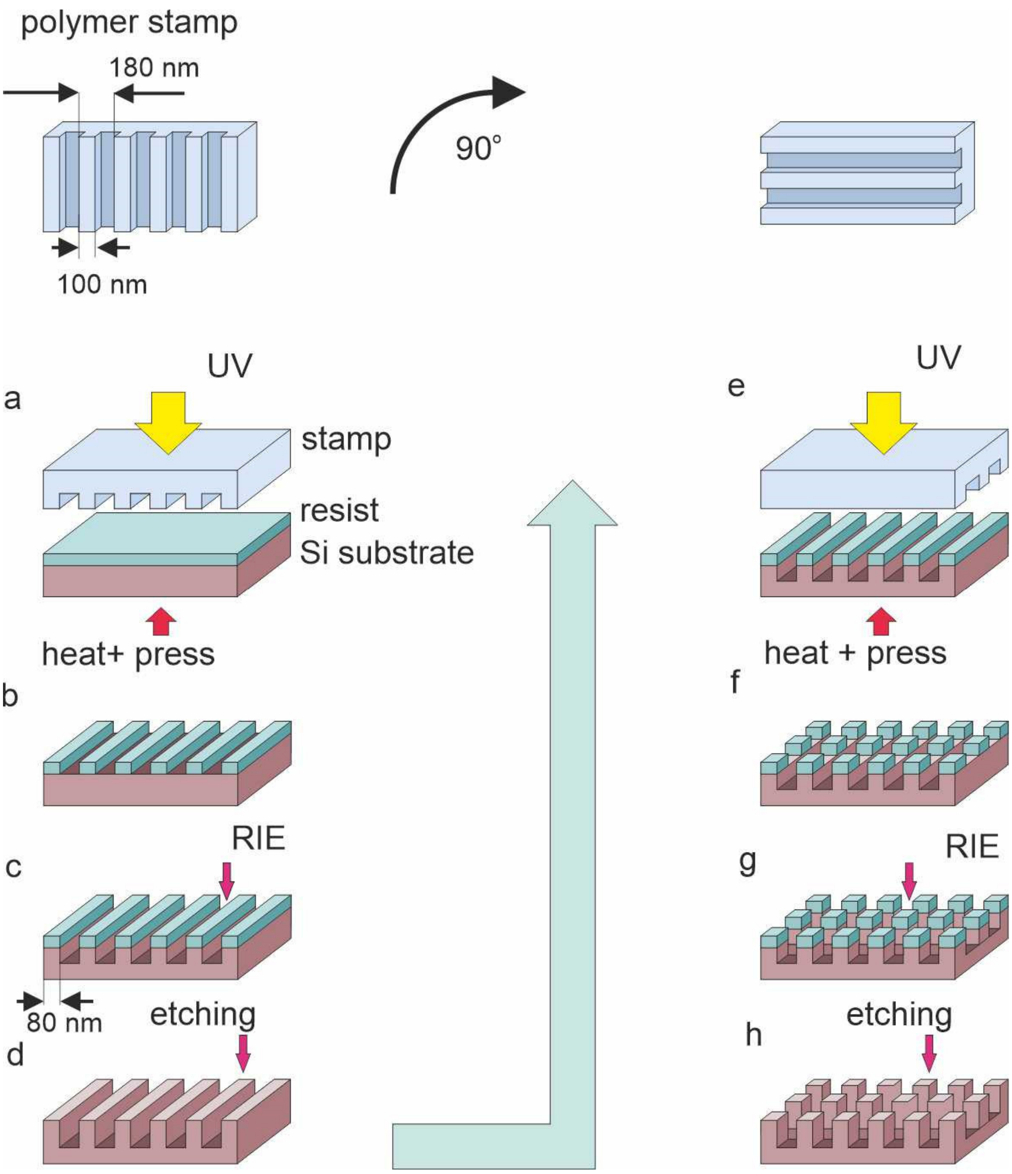

Figure S1. The process of forming the nanostructured surface of a Si substrate using double-stamp nanoimprint lithography and reactive ion etching. After the first lithography and subsequent etching, arrays of periodic strips with a width of 80 nm, a pitch of 180 nm, and a height of 150 nm were formed on the substrate surface. After the second lithography and etching, arrays of square nanopillars with a width of 80 nm, a pitch of 180 nm, and a height of 150 nm were formed on the surface of the Si substrate.

Section S2. XPS survey spectrum of the VO_2 NWs.

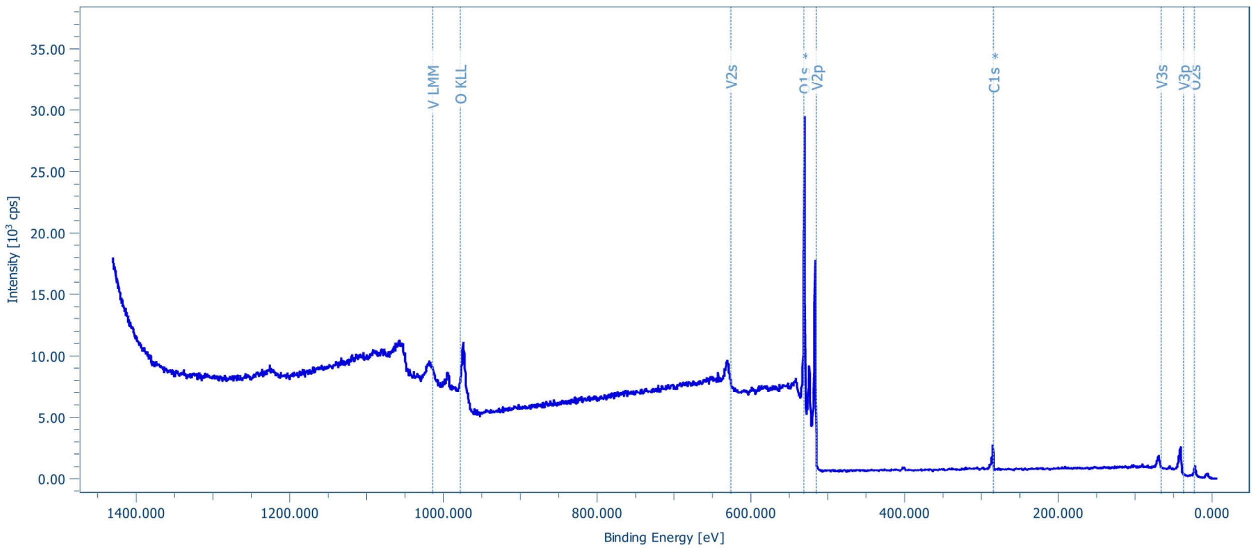

Figure S2. XPS survey spectrum of the VO_2 NWs grown on a nanoimprinted Si substrate.

Section S3. Variation of the surface precursor concentration with the distance from the edge of the sample.

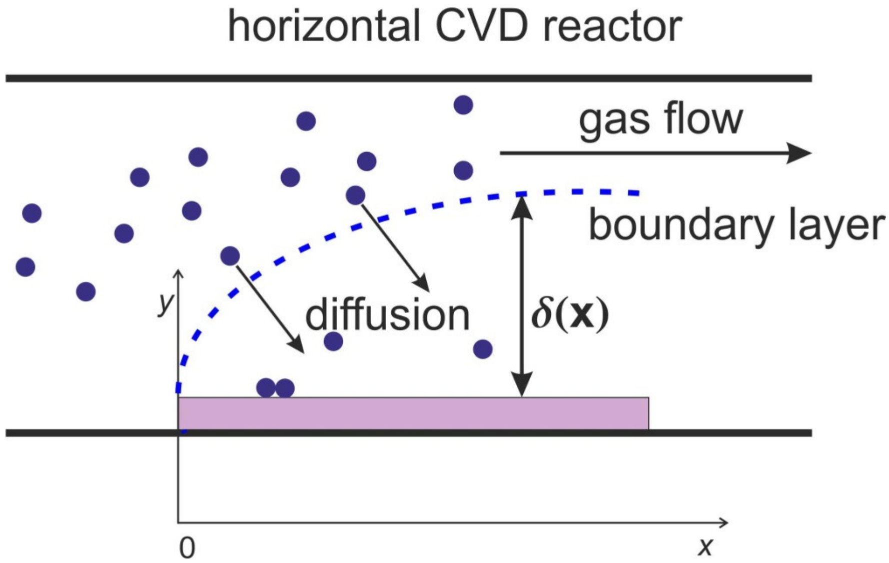

Figure S3. Schematic representation of the diffusion process of precursor particles through the boundary layer in the gas flow. The boundary layer is shown with the dashed blue curve. The dependence δ(x) is the thickness of the boundary layer versus the distance from the edge of the substrate holder (sample).
